# The Response of the Associations of Grass and *Epichloë* Endophytes to the Increased Content of Heavy Metals in the Soil

**DOI:** 10.3390/plants10030429

**Published:** 2021-02-24

**Authors:** Barbara Wiewióra, Grzegorz Żurek

**Affiliations:** 1 Department of Seed Science and Technology, Plant Breeding and Acclimatization Institute-NRI, Radzików, 05-870 Błonie, Poland; 2 Department of Grasses, Legumes and Energy Plants, Plant Breeding and Acclimatization Institute-NRI, Radzików, 05-870 Błonie, Poland; g.zurek@ihar.edu.pl

**Keywords:** endophytic fungi, grasses, grass endophyte interaction, heavy metals, soil pollution

## Abstract

The rapid development of civilization increases the area of land exposed to the accumulation of toxic compounds, including heavy metals, both in water and soil. Endophytic fungi associated with many species of grasses are related to the resistance of plants to biotic and abiotic stresses, which include heavy metals. This paper reviews different aspects of symbiotic interactions between grass species and fungal endophytes from the genera *Epichloë* with special attention paid to the elevated concentration of heavy metals in growing substrates. The evidence shows the high resistance variation of plant endophyte symbiosis on the heavy metals in soil outcome. The fungal endophytes confer high heavy metal tolerance, which is the key feature in its practical application with their host plants, i.e., grasses in phytoremediation.

## 1. Introduction

Technology development and changes in the modern world have a significant impact on the natural environment. Along with the increase of population, demands for energy, consumer goods, machines, devices, etc., are also growing, and this, in turn, affects the environment with pollution from chemicals, pesticides, oils, gasoline, and industrial waste. Contamination of soil with heavy metals and metalloids is currently considered, on a global scale, the main threat to agro-ecosystems, which is associated with food contamination [1].

This is why new solutions are being sought that will allow, in an environmentally friendly way, to eliminate or reduce soil pollution while restoring its productivity. The use of green plants to remove pollutants from the environment to render them harmless (phytoremediation) is the cheapest and the simplest way to use remediation technology [2,3]. However, the use of phytoremediation on a large scale faces some problems, such as slow growth and small biomass, phytotoxicity, and evapotranspiration of volatile contaminants [3,4]. The answer to these problems may come from microbe-assisted phytoremediation in general but with foliar fungal endophytes in particular [2]. The participation of endophytes in the phytoremediation process is based on a positive effect on the growth and development of plants, reducing the phytotoxicity of metals and affecting the translocation and accumulation of metals in the plant.

The total content of chemical pollutants (metals and organic compounds) in soils used for agriculture in Poland does not differ from the levels recorded in neighboring European countries [5]. However, in special cases, i.e., plant cultivation on highly industrialized regions, agriculture lands of high chemicals applications, or in areas close to highways, heavy metals such as lead (Pb), cadmium (Cd), zinc (Zn), nickel (Ni), and copper (Cu) are taken up by plants from the soils [6].

Some of these elements are necessary for the proper functioning of living organisms (e.g., Cu and Zn), while others such as Cd and Pb are the cause of many diseases and disorders. The health effects of systematic consumption of products containing small amounts of heavy metals may become apparent after years due to their cumulative capacity [7]. Heavy metals influence calcium metabolism thereby increasing bone fragility, disturb the functioning of the nervous system causing dementia, intellectual disability, visual disturbances, and coordination of movements, cause damage to the liver and kidneys, and also cause neoplastic changes [7,8].

In soils, heavy metals are commonly found as a result of release from parent rocks in soil-forming processes and during volcanic eruptions [6]. Their natural level does not pose a threat to ecosystems. For example, the average content of heavy metals in agricultural soils of Poland is small and amounts to (mg·kg^−1^), e.g., Cd-0.21, Cu-6.5, Ni-6.2, Pb-13.6, and Zn-32.4 [9]. Over 96% of arable soils are characterized by natural or slightly increased content of heavy metals, which allows us to classify them as high-quality soils, on which all plants can be grown excluding the purpose of growing vegetables for preserves and direct consumption for children [10]. It also follows that over 3% of soils are contaminated with heavy metals to the extent that all grown crops are excluded for consumption. Cationic forms of heavy metals retain in the surface layer of soil and their migration into the soil profile is relatively slow. This is one of the main causes of soil chemical degradation [11]. This type of threat to plant production occurs mainly in industrialized areas and in the vicinity of roads where, together with exhaust fumes, sewage, or industrial dust and heavy metals are collected by plants and introduced in the food chain.

This work aims to present different aspects of the interactions between fungal endophytes in the genus *Epichloë* and their grass host in the response to increased level of heavy metals in soil.

## 2. Heavy Metals

Due to the degree of danger, heavy metals are divided into the following groups [12]:

- very high level of potential threat, e.g., Cd, Hg, Pb, Cu, Zn,
- high level of potential threat, e.g., Mo, Mn, Fe,
- medium level of potential threat, e.g., Ni, Co,
- low level of potential threat, e.g., Sr, Zr.

Heavy metals that are the most common in contaminated sites are Pb, Cr, As, Zn, Cd, Cu, and Hg [13]. Trace metals such as: Cu, Fe, Zn, Mn, Co, and Se are essential for metabolism and while the other ones do not play an important role in the life cycle of organisms (Pb, Cd, Hg, and As). However, they can be damaging to organisms when the toxicity threshold is exceeded [14].

Heavy metals are often essential for the proper functioning of organisms as they perform biochemical and physiological functions. They are important components of several key enzymes and play an important role in various oxidation-reduction reactions [14]. For example, copper is an essential nutrient that is incorporated into a series of enzymes involved in hemoglobin formation, carbohydrate metabolism, catecholamine biosynthesis, and the cross-linking of hair collagen, elastin, and keratin [15]. Zinc plays an important role in the activity of pyrophosphates and in shaping DNA, and iron is part of various enzymes, regulates the functions of chloroplasts or is involved in oxidation processes [16]. Other heavy metals are enzyme activators and take part in photosynthesis (Mo), the formation of chlorophyll (Co) or show antioxidant activity (Se).

In biological systems, heavy metals influence cell organelles and components such as the cell membrane, mitochondria, lysosomes, endoplasmic reticulum, nuclei, and some enzymes involved in metabolism, detoxification, and damage repair [17].

### 2.1. Occurrence in the Soil

The development and extensive application of plant protection chemicals in agriculture, mineral and organic fertilizers, e.g., compost and sewage sludge produced from municipal waste, has led to the excessive accumulation of heavy metals in soils and then in plants. Intensive use of transportation routes emits large quantities of harmful substances into the environment. The main components of traffic pollution are nitrogen oxides, sulfur, polycyclic aromatic hydrocarbons, and heavy metals, such as Pb, Cr, and Cd [18]. A major source of lead is petrol and sources of cadmium include vehicle construction components, tires, or moisturizers. Chrome can come from corrosive parts of metal vehicles [19]. The highest concentrations of heavy metals are recorded in the so-called street dust, collected from the surface directly in the lane. These values can reach even 15,000 mgꞏkg^−1^ for lead, 196 mgꞏkg^−1^ for chromium, and 72 mgꞏkg^−1^ for cadmium [19,20]. This most often applies to the Far East or Africa, where the increase in the number of transport means occurs very quickly, which is not covered by the applicable regulations, which are changed more slowly.

Over 90% of the total content of cadmium, copper, zinc, and lead in soils and sediments of rivers and other water reservoirs comes from anthropogenic pollutants [21]. A particularly high content of heavy metals in the soil was found in the regions adjacent to the smelters of copper [22,23,24,25,26,27,28], of zinc smelters [29,30,31,32,33] and mines [34,35]. Thus, soil pollution of heavy metals has a local character and mainly concerns the areas near the smelters, the landfills of slag or mines where long-term emission of gaseous pollutants and metal-bearing dusts is the source of the enrichment of soils with metals around the emitters of pollutants.

The main carriers of heavy metals were previously active substances of pesticides (e.g., arsenic, copper, mercury, zinc, or lead compounds) [36], but they have since been replaced with different types of organic compounds. Concerning the content of heavy metals in the soil environment, commonly the term “background” or “baseline” are often used as synonymously [18,37]. The natural abundance of an element in rock, sediment, or soil with reference to a particular area is the most common definition of background, and will be used in this meaning in below article.

Higher values from the background are considered as evidence of environmental pollution. European countries have several approaches to define the risk level associated with different concentrations of heavy metal in soil [38,39]. For example, for lead content in soil, background values in Poland are 20–40 mg·kg^−1^, for cadmium-0.05–0.7 mg·kg^−1^ and for chromium-15–60 mg·kg^−1^ [19]. The guideline value standards of the Ministry of Environment of Finland [40] in this regard for lead-60 mg·kg^−1^, cadmium-1 mg·kg^−1^, and chromium-100 mg·kg^−1^. The Finnish standard values represent a good approximation of the mean values of different national systems in Europe [38] and India [41] and they have been applied in an international context for agricultural soils as well [42].

However, we must be aware that the few metals, e.g., Cu, Zn, Co, and Fe in trace amounts are essential for various metabolic activities of plants. However, excess of all kinds of metals (both, essential and non-essential) adversely affect plant metabolism [43]. The acceptable concentrations of heavy metals in the soil are different for individual countries and designated by relevant state offices. For example in Poland limit values for individual metals depend on the soil or soil quality standard and its current and planned function (Table 1).

Agricultural land on which increased heavy metal content has been found should be excluded from cultivation for consumption purposes. It should be proposed to introduce such crops that would not lead to soil degradation (e.g., due to erosion) and would allow the systematic binding of harmful elements.

### 2.2. The Importance of Heavy Metals for Plants, Animals, and Humans

Heavy metals are common in soils. Their natural level does not pose a threat to ecosystems. However, the concentration above the permissible norms already means a serious problem. Through plants, heavy metals enter the human body, where they can contribute to the occurrence of many diseases, including cancer [8,44,45,46,47].

The metals in the form of free ions are most easily absorbed by plants from the soil, while metals in the form of complexes can be mobilized by active substances secreted by plant roots and then taken up by plants [48,49]. The uptake of heavy metals by plants depends on the type of metal, their content in the soil, the forms in which they occur, and the plant species [50]. The heavy metal content of various plant organs is reduced in the following order: root, leaves, stem, flowers, and seeds. The toxic effect of heavy metals on most plants often occurs only in soil with high levels of contamination. On the other hand, humans are particularly sensitive to the presence of increased amounts of heavy metals, especially cadmium and lead (Table 2) [51]. Heavy metals in animal and humans primarily cause changes in protein synthesis and disturbances in ATP production, which may cause serious pathological changes, including cancer [12].

Heavy metals are systemic toxicants known to induce adverse health effects in humans. Exposure to toxic metals causes long-term health problems, mainly cardiovascular disease, developmental abnormalities, neurological and neurobehavioral disorders, diabetes, hearing loss, hematological and immune disorders, and various types of cancer. The severity of the adverse health effects is related to the type of heavy metal and its chemical form, and is also time and dose dependent [52]. The results of studies on the effects of heavy metals on human health indicate that simultaneous exposure to several heavy metals produced more severe effects even at lower dose levels than for one metal [53]. Nordberg et al. [54] showed that simultaneous human exposure to cadmium and inorganic arsenic caused more pronounced kidney damage than exposure to each of these elements separately.

In crop and wild species, there is considerable variability in the ability to accumulate different metal ions from contaminated soils. Within plant species, genotypes characterized by increased ability to accumulate a given metal can be distinguished. In general, plants can grow on land with the content of most heavy metals two or even three times exceeding the limit values. The growth and development of the plant stops in the stage of a few-leafed seedling, only after reaching the content of metals in the order of (in mg·kg^−1^) ca. 1400 Pb, 90 Cd, or over 4500 Zn [65]. These exceedances of heavy metals are, however, very rare and most often encountered in the vicinity of non-ferrous metallurgy plants.

Some plant species, called metallophytes, have the ability to grow and develop on metal-rich habitats. These plants possess the special mechanisms for coping with higher heavy metal concentrations in soil and are divided into three categories: excluders, indicators, and hyperaccumulators [6]. Hyper accumulative plants accumulate heavy metals in their tissues in an amount of metals, often exceeding their concentration in the soil [66]. During the search and selection crop genotypes for phytoremediation, the greatest number of candidates was found among *Brassicaceae*, *Poaceae*, and *Fabaceae* plants. In the grass family, the commonly grown maize is most useful in the phytoextraction process. Other species and varieties in this family come from the genera *Festuca*, *Agrostis,* and *Lolium*. Although they accumulate much less biomass, the cost of their cultivation is much lower and they have lower soil requirements. It seems a good idea to use *Agropyron repens* L., which accumulates the largest amounts of toxic substances in the soil in its roots [67].

So far, research has focused on endophytes, fungal symbionts of grasses, associated with hyperaccumulators because of their resistance to metals associated with long-term adaptation to high metal concentrations in plants [68]. Many such endophytes have been isolated from hyperaccumulating plants, such as *Alyssum bertolonii*, *Alnus firma*, *Brassica napus*, *Nicotiana tabacum*, *Thlaspi caerulescens*, *Thlaspi goesingense*, and *Solanum nigrum* [2].

Plant growth in contaminated soils is usually associated with the accumulation of these elements in their biomass, which in turn significantly limits its further use. The suitability of such biomass for energy conversion is related to the reduction of metal emissions in the energy production process and the management of leftovers after processing (e.g., ashes after combustion, post-concession deposit). The presence of heavy metals for plants can be critical especially in metabolic processes that can be inhibited (Table 3).

However, the presence of some heavy metals is necessary for the course of some life processes, e.g., photosynthesis. Both deficiency and elevated concentration of heavy metals affects both light and dark reactions of photosynthesis directly or indirectly. For example, iron is necessary for the synthesis of chlorophyll, and manganese is required as a cofactor for several enzymes involved in photosynthesis, particularly decarboxylase and dehydrogenase enzymes [69].

## 3. The Symbiosis of Grass-*Epichloë*

### 3.1. Occurrence and Importance

Endophytic fungi are species of the genus *Epichloë*, which live their whole life in symbiosis in the tissues of their hosts plants. The most common grass endophytes include: *E. festucae* var. *lolii* co-living with *L. perenne*, *E. coenophiala* co-occurring with tall fescue (*F. arundinacea*), and *E. uncinata* occurring in meadow fescue (*F. pratensis*) [71]. Previously, the *Epichloë* genus contained only sexual forms (teleomorph), but now also includes asexual forms (anamorph), which were previously classified as *Neotyphodium* [72], and before that, *Acremonium* [73] (Table 4).

Many studies indicate the frequent presence of these fungi in grass plants, both cultivars and wild ecotypes [86,105,106,107,108,109], as well as in the seeds of commercially available varieties [110]. However, maintaining the viability of endophytes in seeds varies depending on the storage conditions, and at the same time it is possible to eliminate them from the seed when necessary, e.g., due to the production of harmful alkaloids. It should be mentioned that seed is not the only source of endophytes spreading, but they can migrate from plant to plant during mowing (i.e., horizontal transmission) [111].

### 3.2. The Effects of Epichloë Endophytes on Plants

The effects of this symbiosis can be both negative and positive. Endophytes confer benefits to grasses, such as mechanisms of tolerance to drought and regeneration of damage after long-term drought, as well as the more effective management of nitrogen and improved phosphorus assimilation [112]. In addition, grasses infected by endophytes are resistant to pests, nematodes, and certain diseases [105]. However, the presence of endophytes in plants can pose a threat to herbivores. It has been found that some of the alkaloids produced by these fungi are toxic and that their accumulation in feed can affect the health status of farm animals [113]. In cattle is fed with grass containing ergovaline or lolitrem B, there may be a decrease in milk production, a decrease in body weight, a decrease in activity (animals are not active, and avoid the sun). In extreme cases, severe diseases such as “ryegrass staggers syndrome” and “fescue toxicosis” can occur [105]. Previous studies have shown that endophytes induce drought tolerance mechanisms in grass plants; however, these effects appear to depend on the species, variety, and developmental phase of plants [107,114]. This favorable aspect of the presence of endophytic fungi in grasses has been best documented for tall fescue [115,116,117,118,119]. In other grass species, e.g., perennial ryegrass, the results of drought resistance studies are not so clear [120,121,122,123]. Gundel et al. [124] indicates that the results obtained in most of the studies on the impact of endophytes on drought stress are inconclusive, often they do not describe any experiments on drought or related information. They argue that there is an unreasonable extrapolation that attributes the overall positive effect of fungal endophytes on drought tolerance of host plants in general and in the long term, and that it has spread in the literature to predict the effects of endophytes on wild species in natural, arid ecosystems. It has also been found that endophytes affect the nitrogen management by both assimilatory and basic nitrogen metabolism and may be correlated with mechanisms of its utilization by endophyte mycelium and better phosphorus assimilation [112], as well as improve the viability of seedlings and tiller number [125]. In addition, grass inhabited by endophyte are resistant to pests, nematodes, and some diseases such as tall fescue with increased resistance to fungus *Rhizoctonia zeae* [105,123,126].

Pressure exerted on plants by disease factors is the most important abiotic stress that grasses are subjected to during the entire growing season. The most dangerous pathogens of perennial ryegrass in turf maintenance are pink snow mold (*Microdochium nivale*), powdery mildew (*Erysiphe graminis*), leaf spot (*Drechslera* spp. and *Bipolaris* spp.), stem rust (*Puccinia graminis*), brown patch (*Rhizoctonia* spp.), and crown rust (*Puccinia coronata*) [127]. The ability to control plant diseases by infecting more desirable *Epichloë* endophytes, which did not affect flowering, was investigated in vitro by White and Cole [128] and Siegel and Latch [129]. They showed that *Epichloë* isolates grown on agar plates inhibited the growth of colonies of many pathogenic fungi including *Rhizoctonia solani*, *R. zeae*, *Bipolaris sorokiniana*, and *Colletotrichum graminicola*. Siegel and Latch [129] also found that the antifungal activity of endophyte differed between strains. Often for many microorganisms there is a low correlation between the effect of antibiosis between fungal cultures under laboratory conditions and the effect on the disease in the field [130]. It was confirmed in field experiments conducted by Burpee and Bouton [131], which showed that the presence of endophytes did not protect tall fescue plants against *R. solani*, the main pathogen occurring in soil. Moreover, endophyte-infected tall fescue seedlings were not protected against *B. sorokiniana* infection [132] and pathogens frequently observed on lawns: *Magnaporthe poae* and *Pythium aphanidermatum* [133]. The presence of endophyte in tall fescue or perennial ryegrass does not appear to affect the occurrence or severity of root rot caused by *Fusarium oxysporum*, *F. equiseti*, and *Pythium acanthicum*, as reported by Hume et al. [134].

Studies on abiotic factors such as water deficit, mowing, shading, low nitrogen fertilization, or low soil pH have demonstrated varying plant responses, depending on the plant genome–endophyte genotype structure [135,136,137]. It was found that the stress tolerance conferred by some endophytes involves also habitat-specific fungal adaptations, and this concept has been confirmed with different fungal and plant species, and different environmental stresses [138]. Redman et al. [139] studied a plant species *Dichanthelium lanuginosum* from the geothermal soils colonized by one dominant endophyte *Curvularia protuberata*. They found that this fungus confers heat tolerance to the host plant, and neither the fungus nor the plant can survive separate from one another when exposed to heat stress more than 38˚C. A comparative study was done by Rodriguez et al. [140] which revealed that the ability to confer heat tolerance was specific to isolates from geothermal plants hence and the ability to confer heat tolerance is a habitat-adapted phenomenon.

In the variable European climatic and soil conditions, the beneficial effects of endophytes in grasses compared to plants without these fungi are not clear. There are reports of plants with an endophyte that have accumulated higher amounts of metals from the soil and even used them to produce more biomass [141,142,143,144]. Our preliminary research shows the possibility of increasing the uptake of cadmium, for example by 25% in plants infected by endophyte versus non-infected plants [145]. In an abiotic and biotic stress conditions, major losses in plant yield take place. Throughout the life cycle, plants can adjust their physiology and metabolism, including the synthesis of a range of defensive proteins to overcome the stress [146]. Further, endophytes that colonize plants can improve plant growth and health through better mineral absorption or increased resistance to biotic and abiotic stresses [147].

## 4. Response of the Endophyte—Grass Association on the Heavy Metals in the Soil

The results of many scientific studies indicated that heavy metals are toxic to plants, but many plants are metal tolerant and some of them are metal hyperaccumulators [148,149,150]. Irdis et al. [68] found that the plants with metal hyper-accumulative properties and associated with endophytes could be metal resistant, due to long-term adaptation to the high concentration of metals accumulated in the plants. The phytoremediation efficiency of contamination of heavy metals is mainly dependent on metal uptake and accumulation in shoots. It has been demonstrated that some heavy-metal resistant and plant-growth-promoting endophytes can improve uptake and accumulation in plants of nickel [151], cadmium [152], or lead [153].

Results of studies [152,154,155,156,157] confirm the key role of endophytes in the adaptation of plants to the conditions of a polluted environment by immobilizing contaminants in the soil, promoting plant growth, decreasing phytotoxicity, and improving plants’ metal tolerance. Some endophytic bacteria can produce indole-3-acetic acid, which stimulates plant growth and enhances phytoremediation [158], or affects the plant’s root elongation compared to plants in which these bacteria were not observed [159]. Other endophytes can produce cytokinins or gibberellins, which under both stress and non-stress conditions can stimulate plant growth and modify their morphology [160]. Many authors [158,161,162] also claim that some of the endophytes exhibit features that can alter contaminants’ toxicity for example through the production of iron chelators, siderophores, organic acids, and various degrading enzymes.

Many studies on the use in the phytoremediation process have been made for endophytic bacteria [163,164,165,166] but also among the fungi, some species have been found that can be successfully used in this process [167,168,169]. Soleimani et al. [167] investigated the effect of two endophyte-infected grass species (*Festuca arundinacea* and *Festuca pratensis*) on the degradation of petroleum hydrocarbons. They found that endophyte-infected plants contained more root and shoot biomass and created higher levels of water-soluble phenols and dehydrogenase activity in the soil. Song et al. [86] show that *Epichloë* endophytes conferred stress tolerance to native grasses in China and played a significant role in the survival of some plants in high-stress environments, such as cadmium contaminated soils. *Achnatherum inebrians* [170,171] and *Elymus dahuricus* [172] had higher germination rates, more tillers, longer shoots and roots, and more biomass compared to endophyte-free plants in high concentration of cadmium ions. There was no significant difference between endophyte-infected and endophyte-free plants under low cadmium concentration, indicating that *Epichloë* infection was only beneficial to the growth and development of *A. inebrians* and *E. dahuricus* exposed to the high concentration of cadmium ions. Further, endophytic fungi inhabiting perennial grasses were the subject of research, the results of which showed that they can be successfully used in the process of phytoremediation [173,174]. 

### 4.1. Aluminum

Malinowski and Belesky [175] reported that *Epichloë coneophialum* infection had no effect on root and shoot development in tall fescue (*Festuca arundinacea*) grown in elevated concentration of Al. A higher concentration of Al (35%) and P (10%) has been observed in E+ plants, but no differences were found in roots. Elevated concentration of Al gave a variable effect on root and shoot dry weight (from positive to negative) in red fescue (*Festuca rubra*) and *Poa ampla* inhabited by *Epichloë festucae* and *Epichloë* sp., respectively [176]. However they claim that endophyte infection alone is not enough to confer aluminum tolerance in fine fescues, but in certain plant–fungus combinations, endophyte infection can contribute to enhanced aluminum tolerance.

### 4.2. Cadmium

Cadmium (Cd) is one of the most toxic environmental pollutants for plants [177]. Cadmium can interfere with numerous biochemical and physiological processes including photosynthesis, respiration, nitrogen and protein metabolism, and nutrient uptake [178].

Generally, the presence of endophytes had a positive effect on plants grown in elevated Cd concentrations. *Epichloë* endophyte presence in the seed of *Elymus dahuricus* promoted germination range and index, as compared to endophyte-free seed, in higher (i.e., from 100 to 300 μmol L^−1^) Cd concentrations [172]. Higher values of shoot and root length and dry biomass were also observed in E+ plants of mentioned grass species.

Presence of endophytes in tall fescue (*Epichoë* sp.) and *Achnatherum inebrians* (*Epichloë gansuensee*) grown in the presence of an elevated concentration of Cd yielded also increased tiller number and higher biomass [167,179]. Plants inhabited by endophytes were able to accumulate more Cd ions in roots and shoots [167,179]. This is the result of improved transport of Cd ions from roots to shoots and resulting higher (2.4-fold) phytoextraction efficiency of E+ plants [179]. The effects of cadmium on germination, and antioxidative enzyme activity present within *Achnatherum inebrians*, were determined for plants infected and non-infected by *Neotyphodium gansuense* [171]. They found that under high Cd concentrations, endophyte-infected plants exhibited a higher germination rate and index, and higher values for shoot length, root length, and dry biomass, but there was no significant difference under low Cd concentrations. Endophyte infection was concluded to be beneficial to the germination and anti-oxidative mechanisms within *A. inebrians* under plant exposures to high cadmium concentrations. In the case of this plant increased anti-oxidative enzyme activity, H_2_O_2_ content and increased levels of chlorophyll *a* and *b*, as well as declined proline and malondialdehyde content, were observed in E+ plants vs. E- plants [172]. All these results indicate that the presence of endophyte may ameliorate the effect of cadmium toxicity on plant.

### 4.3. Copper

Copper (Cu) is an essential element to plant growth but toxic in higher concentrations. The presence of endophyte (*Epichloë coenophialum*) in tall fescue was associated with lower Cu concentrations in plants, which may further contribute to lowered Cu status in grazing animals, thus contributing to the etiology of fescue toxicosis [180]. However, plants were grown on pasture or pots without artificially increased Cu levels, which was one from many micronutrients in soil. Other results suggest that the effects of endophyte infection on Cu acquisition in perennial ryegrass (*Lolium perenne*) is quite different from those found in tall fescue [181]. This was explained that *Epichloë* endophytes present in perennial ryegrass often triggers sets of physiological, biochemical, and morphological responses in host plant different from those found for tall fescue infected with *N. coenophialum* [112]. To our knowledge, no research is available concerning the effect of artificially increased, high concentrations of Cu in growing medium on the grass performance and endophyte interaction. Most of recent research concerns natural Cu deficiency in forage where endophyte-induced decrease of Cu in stems and leaves was an additional negative effect for animals.

### 4.4. Nickel

Studies of Mirzahosseini et al. [182] have shown that endophytes also affect the presence of nickel in the soil. The obtained results indicated that some E+ genotypes were characterized by better growth and tolerance to this chemical element. However, the results were inconclusive, because otherwise endophyte free genotypes showed greater tolerance to Ni stress compared to E + plants. This revealed that the effect of endophyte infection in *Festuca* plants depends on the host genotype [182].

### 4.5. Zinc

Endophytes infecting tall fescue and perennial ryegrass have been found to improve chlorophyll functions (i.e., Fv/Fm) under high concentrations of Zn in growing medium [174]. Shoots of endophyte-infected tall fescue had 82% greater concentration of Zn than endophyte-free plants. The opposite relation was found for perennial ryegrass plants: endophyte-free plants exposed higher (29%) concentration of Zn in shoots than endophyte-infected. Other authors suggested no statistically significant differences between endophyte-free and endophyte-infected plants of tall fescue in Zn content in plant tissues [141].

The increase in zinc concentration induced some reduction in photosystem II (PSII) activity of perennial ryegrass plants but not enough to account for the total drop in the net photosynthetic rate [143]. In plants with endophyte, the quenching of the reaction center and antenna complexes rose simultaneously and at a constant rate, as zinc concentrations increased. The same authors examining the concentration of Zn in the leaves stated that it increases with the increase in Zn in the medium and the number of days after which this study was carried out. At the same time, they found that the concentration of Zn in the leaves was 24–32% lower in the presence of *N. lolii* [142]. Leaf dry weight was higher in the presence of endophyte, particularly at 5 mM Zn. It is a known fact that the effect of endophyte for its host is the production of a greater number of tillers [183]. It has been confirmed by Monnet et al. [144] for Zn stress from 1 to 10 mM. Zamani et al. [174] have shown that the number of plant tillers grown under different Zn concentrations was greater in endophyte-infected *Festuca* and *Lolium* compared to their endophyte-free plants. Roots and shoots dry weights in infected *Festuca* plants were also greater than non-infected. Endophyte infected *Festuca* and *Lolium* improved chlorophyll fluorescence as Fv/Fm at high concentrations of Zn, showing their better chlorophyll functions and significant reduction of Zn stress in endophyte-infected plants.

Some interactions of fungal endophytes on heavy metals are shown in Table 5.

Weyens et al. [3] claim that endophyte infection not only improves plant growth but also to reduces phytotoxicity of contaminants and increase metal translocation to the above-ground plant parts. Moreover, endophytes that can break down organic pollutants in soil and facilitate the uptake and accumulation of metals may be a way to improve the phytoremediation process [167,185]. However the process of optimal phytoremediation requires the presence of such associations, where endophyte isolates have both tolerance to the accumulation of pollutants and the production of phytohormones, which can also affect plant growth and metabolism [186].

## 5. Conclusions

Endophytes of the genus *Epichloë* are well-known associates of grasses and research shows their potential use in the phytoremediation of areas excluded from agricultural activities due to heavy metal contamination. There are many known associations of grasses with fungi of the *Epichloë* genus, which can be an excellent basis for research on their potential use in areas excluded from agricultural activities due to the increased content of heavy metals.

Grasses inhabited by endophytes are sometimes characterized by a much higher ability to accumulate heavy metals from the soil. The cultivation of grass-endophyte symbiotes in areas contaminated with heavy metals has a positive effect on the quality of the soil, significantly reducing its phytotoxicity. Monnet et al. [142] claim that the action of the endophyte remains unclear, but different mechanisms are possible. The fungus may accumulate heavy metals in mycelia or influencing on the mechanism to the limit metals concentration in the leaves through a restriction in its transport to the leaf tissues [187,188].

In some cases, plants inhabited by endophytes may have ecological and evolutionary advantage over uninfected, but there exist great variability of plant reactions, even concerning the same host species and the same fungus in uniform growth conditions. The choice of most effective grass–endophyte association for phytoremediation should be based on the earlier evaluation of the efficiency of the symbiosis. Summarizing, the fungal endophytes pass high metal tolerance capacities suggesting their future utilization in endophyte-assisted phytoremediation applications [189].

## Figures and Tables

**Table 1:** Permissible levels of substances (in mgꞏkg^−1^ of dry soil, soil surface layer: 0–0.2 m) causing risk for given land categories based on the Annex to the Regulation of the Minister of Environment from 1 September 2016).

Metal	Land Categories *					
	I	II			III	IV
		II-1	II-2	II-3		
**Arsene (As)**	**25**	10	20	50	50	100
Bar (Ba)	400	200	400	600	1000	1500
Chrome (Cr)	200	150	300	500	500	1000
Zinc (Zn)	500	300	500	1000	1000	2000
Cadmium (Cd)	2	2	3	5	10	15
Cobalt (Co)	50	20	30	50	100	200
Copper (Cu)	200	100	150	300	300	600
Molybdenum (Mo)	50	10	25	50	100	250
Nickel (Ni)	150	100	150	300	300	500
Lead (Pb)	200	100	250	500	500	600
Mercury (Hg)	0.5	2	4	5	10	30

* Land categories: I-residential and other built-up areas, developed agricultural land, recreational and leisure areas, areas with entertainment functions, such as: amusement parks and amusement parks, zoological and botanical gardens; water intakes and their protection zones. II-arable land and areas of family allotment gardens arranged on orchards, permanent meadows, pastures, land under ponds and ditches and areas of family allotment gardens arranged on land; subdivided into following 3 groups: II-a: very light and light mineral soils, granulometric fraction (GF) below 0.02 mm between 10- and 20%); II-b mineral soil from light to heavy, GF 0.02 mm between 10- and 35%, mineral-organic soil with 3.5–6.0% of C org.; II-c mineral and mineral-organic soils of GF 0.02 mm above 20%, pH higher than 5.5, more than 6.0% of C org. National parks and nature reserves. III-forests, wooded and shrubby land, wastelands, recreational and leisure areas of a historic nature (castle ruins, fortified settlements, burial mounds, natural monuments), unmanaged green areas, ecological lands. IV-industrial and urbanized areas, communication areas (roads, and railway areas).

**Table 2:** The most important heavy metals and their toxic effect on humans and animals (more details: Tchounwou et al. [52], Siemiński [55], Ware [56], Walker et al. [57], Kołacz and Brodak [58], Szymański [59], Zwoźniak [60], Brandys [61], Albińska et al. [62], Hong et al. [63], Davidson et al. [64]).

Metal	Effect	Accumulation
Cadmium	- disturbs protein metabolism - interferes with the transformation of vitamin B1 - affects metabolism of calcium and phosphorus compounds - increases bone fragility - carcinogenic, embryotoxic and teratogenic - anemia, hypertensive disease, changes in the circulatory system, pregnancy complications - changes in pulmonary function, increased risk of emphysema - Itai-itai disease	- liver, kidneys, pancreas, intestines, lungs
Lead	- poisoning of the digestive, nervous, blood, respiratory, kidney and liver systems - embryotoxic, carcinogenic and mutagenic effects - disorders of reproductive functions and calcium metabolism - impaired development, lower IQ, shortened attention span, hyperactivity, and mental deterioration	- skin and brain, - blood, where it reaches all parts of the body and accumulates in almost every tissue
Arsenic	- affects virtually all organ systems, including the cardiovascular, dermatological, nervous, gastrointestinal and respiratory system - a relationship with diabetes and reproductive organs	- absorbed into the body and metabolized, expelled mainly through the urine
Zinc	- carcinogenic - metabolic disorders (causes anemia), susceptibility to infections, immune and mental development disorders - disorders of the digestive and respiratory systems	- liver, kidneys
Chrome	- allergic factor - damages the digestive system - carcinogenic, embryotoxic and teratogenic	- liver, kidneys, lung tissue, blood, bone marrow and spleen
Mercury	- brain damage - cell damage - paralyzes sensory nerve endings, - visual, hearing, speech disorder and limb muscle paralysis - severe poisoning of the whole organism and even death.	- kidneys, liver, nervous system

**Table 3:** A negative effect of some heavy metals on plants.

Metal	The Symptoms and Effect	References
Lead	- lower yielding, stunted leaves often with necrotic spots and shortened roots with less hair density, - photosynthesis, cell division, nitrogen metabolism and water management disorders,	- Kabata-Pendias and Mukhejee, 2007 [51]
Cadmium	- chlorotic and brown spots on leaf blades, reddening of veins, twisted leaves, shortening of roots, - disturbances in photosynthesis, transformations of nitrogen compounds, changes in the permeability of cell membranes, changes in DNA structure,	- Kabata-Pendias and Pendias, 2001 [11], Kabata-Pendias and Mukhejee, 2007 [51]
Copper	- growth inhibition, chlorosis, necrosis, and leaf depigmentation, - decrease in fluorescence, - decline in photosynthesis,	- Küpper et al., 1996 [69], Vidaković-Cifrek et al., 2015 [70]
Zinc	- both the deficiency and the excess of this element limit the growth and development of plants, - excess causes the inhibition of plant growth and reproduction, chlorotic and necrotic changes on leaves, reduction of photosynthesis, - the deficiency disturbs the metabolism of proteins, phosphates, carbohydrates and the synthesis of RNA and DNA.	- Kabata-Pendias and Mukhejee, 2007 [51]

**Table 4:** Some *Epichloë* species and their select host.

Grass Species	*Epichloë* Species	Reference
*Achnatherum inebrians*	*E. gansuensis*	Li et al. 2004 [74]
*Achnatherum* sp.	*E.chisosum, E. inebrians, E. funkii*	Moon et al. 2004 [75]; Chen et al. 2015 [76]; Leuchtmann et al. 2014 [72]
*Agropyron repens*	*E. bromicola*	Lembicz et al., 2010 [77]
*Agrostis* spp.	*E. baconii*, *E. amarillans*	Cagnano et al., 2019 [78], Clay and Brown, 1997 [79], White, 1993 [80]
*Ammophila breviligulata*	*E. amarillans*	Drake et al. 2018 [81]
*Anthoxanthum* sp.	*E. typhina*	Cagnano et al., 2019 [78]
*Brachyelyrtum* sp.	*E. brahyelytrei*	Cagnano et al., 2019 [78]
*Brachypodium* sp.	*E. sylvatica*, *E. typhina*	Cagnano et al., 2019 [78]
*Bromus aleuticus*	*E. pampeana; E. tembladare*	Leuchtman et al. 2014 [72]
*Bromus erectus*	*Epichloë bromicola*	Leuchtmann and Schardl, 1998 [82]
*Bromus laevipes*	*E. cabralii, E.* spp.	Charlton et al. 2014 [83]
*Bromus setifolius*	*E. tembladerae*	Leuchtman et al. [72]
*Bromus setifolius*	*E.typhina* var*. aonikenhana*	McCargo et al. 2014 [84]
*Bromus setifolius*	*E. typhina*	Gentile et al. 2005 [85]
*Calamagrostis* sp.	*E. stromatolonga*	Song et al. 2016 [86]
*Cinna arundinacea*	*E. schardlii*	Ghimire et al. 2011 [87]
*Dactylis glomerata*	*E. typhina*	Clay and Brown, 1997 [79]
*Elymus canadensis*	*E. canadensis*	Leuchtman et al. 2014 [72]
*Elymus repens*	*E. elymi, E. bromicola*	Cagnano et al., 2019 [78], Leuchtmann and Schardl, 1998 [82]
*Festuca argentina*	*E. tembladerae*	Cabral et al. 2007 [88]
*Festuca brevipila*	*E. festucae*	Clarke et al. 2006 [89]
*Festuca arizonica*	*E. huerfanum, E. tembladare*	Moon et al. 2004 [75]
*Festuca arundinacea*	*E. coenophialum*	Cagnano et al., 2019 [78]
*Festuca gigantea*	*E. festucae*	Leuchtmann et al., 1994 [90]
*Festuca hieronymi*	*E. tembladerae*	Cabral et al. 2007 [88]
*Festuca longifolia*	*E. festucae*	Niones and Takemoto 2014 [91]
*Festuca pratensis*	*E. uncinatum, E. siegelii*	Craven et al., 2001 [92], Gams et al., 1990 [93]
*Festuca pulchella*	*E. festucae*	Niones and Takemoto 2014 [91]
*Festuca rubra*	*E. festucae*	Clay and Brown, 1997 [79], Leuchtmann et al., 1994, [90]
*Festuca sinensis*	*E.* sp.	Zhou et al. 2015 [94]
*Festuca* sp.	*E. sinofestucae*	Song et al. 2016 [86]
*Glyceria* sp.	*E. glyceriae*	Schardl and Leuchtmann 1999 [95]
*Holcus lunatus*	*E. clarkii*	Clay and Brown, 1997 [79], Leuchtmann et al., 2014 [72]
*Holcus* *mollis*	*E. mollis*	Clay and Brown,1997 [79], Morgan- Jones and Gams, 1982 [73]
*Hordelymus* sp.	*E. disjuncta, E. danica, E. hordelymi, E. sylvatica* subsp*. pollinensisi*	Leuchtmann and Oberhofer, 2013 [96]; Leuchtmann et al., 2014 [72]
*Hordeus comosum*	*E. tembladerae; E.amarillans; E. typhina hybrids*	Iannone et al. 2015 [97]
*Koeleria cristata*	*E. festucae*	Niones and Takemoto 2014 [91]
*Leymus chinensis*	*E. bromicola*	Wang et al., 2016 [98]
*Lolium canariense*	*E. typhinum* var*. canariense*	Moon et al., 2000 [99]
*Lolium multiflorum*	*E. occultans*	Moon et al., 2000 [99]
*Lolium perenne*	*E. festucae* var*. lolii, E. typhina, E. lolii, E. hybrida*	Latch et al., 1984 [100], Morgan-Jones and Gams, 1982 [73]
*Lolium rigidum*	*E. occultans*	Leuchtmann et al., 2014 [72]
*Melica ciliata*	*E. guerinii*	Leuchtman et al. 2014 [72]
*Melica decumbens*	*E. melicicola*	Moon et al. 2004 [75]; Moon et al. 2002 [101]
*Phleum alpinum*	*E. tembladerae*	Leuchtman et al. 2014 [72]
*Phleum alpinum*	*E. cabralii*	McCargo et al. 2014 [84]
*Phleum* sp.	*E. typhina*	Cagnano et al., 2019 [78]
*Poa alsodes*	*E. alsodes*	Shymanovich et al. 2017 [102]
*Poa secunda* ssp. *junicolia*	*E. poae*	Tadych et al., 2012 [103]
*Poa* spp.	*E. typhina, E. typhina* subsp*. poae,*	Tadych et al., 2012 [103]
*Poa* spp.	*E. liyangensis*	Li et al. 2006 [104]
*Roegneria* sp.	*E. sinica, E yangzii*	Song et al. 2016 [86]; Li et al. 2006 [104]
*Sphaenopholis* sp.	*E. amarilians*	Cagnano et al., 2019 [78]

**Table 5:** Grass–fungal endophyte association and their influence on heavy metals.

Host	Fungi	Effect	References
*Achnatherum inebrians*	*Epichloë gansuensis* (=*Neotyphodium gansuense*)	better germination rates and index in the high concentration of cadmium, increased tolerance to cadmium by improving the antioxidant defense system	Zhang et al. 2010a [170], Zhang et al. 2010b [171]
*Elymus dahuricus*	*Epichloë* spp. (=*Neotyphodium* spp.)	positively affected seed germination and seedling growth exposed to high Cd concentration	Zhang et al. 2012 [172]
*Festuca arundinacea*	*Epichloë coenophiala* (=*Neotyphodium coenophialum*)	increased exudation of phenolic-like compounds from roots improved Al tolerance	Malinowski and Belesky 1999 [175]
*Festuca arundinacea, F. pratensis*	*Epichloë* spp.	improved cadmium tolerance and bioaccumulation and showed better germination potential	Soleimani et al. 2010a [167]
*Festuca arundinacea, Lolium perenne*	*Epichloë* spp. (=*Neotyphodium* spp.)	accumulation and transport more Zn in aboveground parts under Zn-stress, a significant effect on the photochemical efficiency of photosynthesis	Zamani et al. 2015 [174]
*Lolium arundinaceum* (*Festuca arundinacea*)	*Epichloë coenophiala* (=*Neotyphodium coenophialum*)	enhanced Cd accumulation in plant and improved its transport from the root to the shoot	Ren et al. 2011 [184]
*Lolium perenne*	ryegrass endophytes	increase Cd transport and accumulation in shoots	Ren et a. 2006 [179]
*Lolium perenne*	*Epichloë* spp.	the increase of accumulation of cadmium and copper in aerial parts of the host plants, better plant growth and photosynthesis in the elevated concentration of Cu	Żurek et al. (in press) [145]
*Lolium perenne*	*Epichloë lolii* (=*Neotyphodium lolii*)	a limitation of the Zn concentration in the leaves	Monnet et al. 2001 [142]

## Data Availability

Not applicable.

## References

[1] Kabata-Pendias A., Szteke B. (2012). Pierwiastki Śladowe w Geo- i Biosferze.

[2] Li H.Y., Wei D.Q., Shen M., Zhou Z.P. (2012). Endophytes and their role in phytoremediation. Fungal Divers.

[3] Weyens N., van der Lelie D., Taghavi S., Vangronsveld J. (2009). Phytoremediation: Plant-endophyte partnerships take the challenge. Curr. Opin. Biotechnol..

[4] Gerhardt K.E., Huang X.D., Glick B.R., Greenberg B.M. (2009). Phytoremediation and rhizoremediation of organic soil contaminants: Potential and challenges. Plant Sci..

[5] Maliszewska-Kordybach B., Smreczak B., Klimkowicz-Pawlas A. (2013). Zagrożenie zanieczyszczeniami chemicznymi gleb na obszarach rolniczych w Polsce w świetle badań IUNG-PIB w Puławach. Studia I Raporty IUNG-PIB. Zeszyt.

[6] Ali H., Khan E., Ilahi I. (2019). Environmental and ecotoxicology of hazardous heavy metals, environmental persistence, toxicity, and bioaccumulation. Hindawi J. Chem..

[7] Järup L. (2003). Hazards of heavy metal contamination. Br. Med. Bull..

[8] Sinicropi M.S., Caruso A., Capasso A., Palladino C., Panno A., Saturnino C. (2010). Heavy metals: Toxicity and carcinogenicity. Pharmacology.

[9] Woch F. (2007). Wademekum Klasyfikatora Gleb. Praca Zbiorowa.

[10] GIOŚ, Główny Inspektorat Ochrony Środowiska (2014). Stan Środowiska w Polsce. Raport 2014.

[11] Kabata-Pendias A., Pendias H. (1993). Biogeochemia Pierwiastków Śladowych.

[12] Ociepa- Kubicka A., Ociepa E. (2012). Toksyczne oddziaływanie metali ciężkich na rośliny, zwierzęta i ludzi. Inżynieria I Ochr. Środowiska.

[13] USEPA (1996). Report: Recent Developments for In Situ Treatment of Metals Contaminated Soils.

[14] WHO/FAO/IAEA (1996). Trace Elements in Human Nutrition and Health.

[15] Stern B.R. (2010). Essentiality and toxicity in copper health risk assessment: Overview, update and regulatory considerations. Toxicol. Environ. Health A.

[16] Marschner H. (2005). Mineral Nutrition of Hogher Plants.

[17] Wang S., Shi X. (2001). Molecular mechanisms of metal toxicity and carcinogenesis. Mol. Cell Biochem..

[18] Bezak-Mazur E. (2001). Elementy Toksykologii Środowiskowej. Skrypt nr 32.

[19] Christoforidis A., Stamatis N. (2009). Heavy metal contamination in street dust and roadside soil along the major national Road in Kavala’s region, Greece. Geoderma.

[20] Lu X., Wang L., Li L.I., Lei K., Huang L., Kang D. (2010). Multivariate statistical analysis of heavy metals in street dust of Baoji, NW China. J. Hazard. Mater..

[21] Kyzioł J. (1994). Minerały Ilaste Jako Sorbenty Metali Ciężkich.

[22] Kabała C., Singh B.R. (2001). Fractionation and mobility of copper, lead, and zinc in soil profiles in the vicinity of a copper smelter. J. Environ. Qual..

[23] Vorobeichik E.L., Kaigorodova S.Y. (2017). Long-term dynamics of heavy metals in the upper horizons of soils in the region of a copper smelter impacts during the period of reduced emission. Eurasian Soil Sci..

[24] Nikolić D., Milošević N., Živković Ž., Mihajlović I., Kovaćević R., Petrović N. (2011). Multi-criteria analysis of soil pollution by heavy metals I vicinity of the Copper Smelting Plant in Bor (Serbia). J. Serb. Chem. Soc..

[25] Medyńska-Juraszek A., Kabała C. (2010). Heavy metals concentration andextractability in forest litters in the area impacted by copper smelter near Legnica. Ecol. Chem. Eng. A.

[26] Kabała C., Chodak T., Szerszeń L. (2008). Influence of land use pattern on changes in copper content in soils around a copper smelter, based on a 34-year monitoring cycle. Žemėsūkio Mokslai.

[27] Martley E., Gulson B.L., Pfeifer H.R. (2004). Metal concentrations in soils around the copper smelter and surrounding industrial complex of Port Kembla, NSW, Australia. Sci. Total Environ..

[28] Adamo P., Dudka S., Wilson M.J., McHardy W.J. (2002). Distribution of trace elements in soils from the Sudbury smelting area (Ontario, Canada). Water Air Soil Pollut..

[29] Cuske M., Marcinkiewicz M., Szopka K., Karczewska A., Pora E. (2013). The influence of Oława zinc smelter on soil environment of adjacentareas in the light of total content of heavy metals in surface levels of Oława soils. Pap. Uni. Zielona Góra/Environ. Eng. Ser..

[30] Filzek P., Spurgeon D., Broll G., Svendsen C., Hankard P., Kammenga J., Donker M., Weeks J. (2004). Pedological characterization of sites along a transect from a primary cadmium/lead/zinc smelting works. Ecotoxicology.

[31] Goodarzi F., Sanei H., Garrett R.G., Duncan W.F. (2002). Accumulation oftrace elements on the surface soil around the Trail smelter, British Columbia, Canada. Environ. Geol..

[32] Sterckeman T., Douay F., Proixa N., Fourrierb H. (2000). Vertical distribution of Cd, Pb and Zn in soils near smelters in the north of France. Environ. Pollut..

[33] Sterckeman T., Douay F., Proixa N., Fourrierb H., Perdrix E. (2002). Assessment of the contamination of cultivated soils by eighteen trace elements around smelters in the north of France. Water Air Soil Pollut..

[34] Roszyk E., Szerszeń L. (1988). Nagromadzenie metali ciężkich w warstwie ornej gleb stref ochrony sanitarnej przy hutach miedzi. Roczniki Gleboznawcze T. XXXIX.

[35] Pasieczna A. (2002). Zawartość cynku w glebach wybranych miast w Polsce. Zeszyty Naukowe Komitetu “Człowiek i Środowisko”. PAN.

[36] Terelak H., Motowicka-Terelak T., Stuczyński T., Pietruch C. (2000). Pierwiastki Śladowe (Cd, Cu, Ni, Pb, Zn) w Glebach Użytków Rolnych Polski.

[37] Kobierski M., Dąbkowska–Naskręt H. (2012). Local bacgrounds concentration of heavy metals in various soil types formed from glacial till of the Inowrocławska Plain. J. Elem..

[38] Carlon C., D’Alessandro M., Swartjes F. (2007). Derivation methods of soil screening values in Europe: A review of national procedures towards harmonisation. EUR. Scientific and Technical Research Series.

[39] Ferguson C.C. (1999). Assessing risks from contaminated sites: Policy and practice in 16 European countries. Land Contam. Reclam..

[40] (2007). Government Decree on the Assessment of Soil Contamination and Remediation Needs.

[41] Awasthi S.K. (2000). Prevention of Food Adulteration Act No. 37 of 1954. Central and State Rules as Amended for 1999.

[42] UNEP, International Resource Panel (2013). Environmental Risks and Challenges of Anthropogenic Metals Flows and Cycles. A Report of the Working Group on the Global Metal Flows to the InternationalResource Panel.

[43] Hall J.L. (2002). Cellular mechanisms for heavy metal detoxification and tolerance. J. Exp. Bot..

[44] Al-Saleh I., Abduljabbar M. (2017). Heavy metals (lead, cadmium, methylmercury, arsenic) in commonly imported rice grains (Oryza sativa) sold in Saudi Arabia and their potential health risk. Int. J. Hyg. Environ. Health.

[45] Abtahi M., Fakhri Y., Conti G.O., Keramati H., Zandsalimi Y., Bahmani Z., Ghasemi S.M. (2017). Heavy metals (As, Cr, Pb, Cd and Ni) concentrations in rice (Oryza sativa) from Iran and associated risk assessment: A systematic review. Toxin Rev..

[46] Thounwou P.B., Patlolla A.K., Centeno J.A. (2003). Carcinogenic and systemic health effects associated with arsenic exposure-A critical review. Toxicol. Pathol..

[47] Khan A., Khan S., Khan M.A., Qamar Z., Waqas M. (2015). The uptake and bioaccumulation of heavy metals by food plants, their effects on plants nutrients, and associated health risk: A review. Environ. Sci. Pollut. Res..

[48] Chaney R., Malik M., Li Y.M., Brown S.L., Brewer E.P., Angle J.S., Baker A.J.M. (1998). Phytoremediation of soil metals. Curr. Options Biotechnol..

[49] Inal A., Gunes A., Zhang F., Cakmak I. (2007). Peanut/maize intercropping induced changes in rhizosphere and nutrient concentrations in shoots. Plant Physiol. Biochem..

[50] Jin C.W., Zheng S.J., He Y.F., Zhou G.D., Zhou Z.X. (2005). Lead contamination in tea garden soils and factors affecting its bioavailability. Chemosphere.

[51] Kabata-Pendias A., Mukherjee A.B. (2007). Trace Elements from Soil to Human.

[52] Tchounwou P.B., Yedjou C.G., Patlolla A.K., Sutton D.J. (2012). Heavy metals toxicity and the environment. NIH.

[53] Wang G., Fowler B.A. (2008). Roles of biomarkers in evaluating interactions among mixtures of lead, cadmium and arsenic. Toxicol. Appl. Pharmacol..

[54] Nordberg G.F., Jin T., Hong F., Zhang A., Buchet J.P., Bernard A. (2005). Biomarkers of cadmium and arsenic interactions. Toxicol. Appl. Pharmacol..

[55] Siemiński M. (2008). Środowiskowe Zagrożenia Zdrowia.

[56] Ware G.W. (2001). Reviews of Environmental Contamination and Toxicology.

[57] Walker C.H., Hopkin S.P., Sibly R.M., Peakall D.B. (2002). Podstawy Ekotoksykologii.

[58] Kołacz R., Bodak E., Bodak B., Dobrzyński Z. (1997). Toksyczność Metali Ciężkich, [w:] Ekotoksykologiczne Problemy Chowu Zwierząt w Rejonach Skażeń Metalami Ciężkimi.

[59] Szymański K. (2009). Związki ołowiu i chromu w środowisku naturalnym i odpadach. Rocz. Ochr. Środow.

[60] Zwoździak J. (2002). Człowiek, Środowisko, Zagrożenie.

[61] Brandys J. (1999). Toksykologia Wybrane Zagadnienia.

[62] Albińska J., Goralski J., Szynkowska M., Leśniewska E., Paryjczak T. (2011). Rtęć w tłuszczach zwierząt łownych pochodzących z terenu wojewodztwa łodzkiego. Rocznik Ochrona Środow..

[63] Hong Y.S., Song K.H., Chung J.Y. (2014). Health effects of chronic arsenic exposure. J. Prev. Med. Public Health.

[64] Davison A.G., Fayers P.M., Taylor A.J., Venables K.M., Darbyshire J., Pickering C.A. (1988). Cadmium fume inhalation and emphysema. Lancet.

[65] Żurek G., Pogrzeba M., Rybka B., Prokopiuk K., Barth D.M.S. (2012). Suitability of grass species for phytoremediation of soils polluted with heavy-metals. Breeding Strategies for Sustainable Forage and Grass Development.

[66] Baker A.J.M., Brooks R.R. (1989). Terrestrial higher plants which hyperaccumulate metallic elements–A review of their distribution, ecology and phytochemistry. Biorecovery.

[67] Piotrowska-Niczyporuk A., Bajguz A., Ciereszko I., Bajguza A. (2013). Fitoremediacja-alternatywa na czyste środowisko. Różnorodność Biologiczna–od Komórki do Ekosystemu. Rośliny i Grzyby w Zmieniających się Warunkach Środowiska.

[68] Idris R., Trifonova R., Puschenreiter M., Welzel W.W., Seissitsch A. (2004). Bacterial communities associated witch flowering plants of the Ni hyperaccumulator *Thlaspi goesingenes*. Appl. Environ. Microbiol..

[69] Küpper H., Küpper F., Spiller M. (1996). Environmental relevance of heavy metal substituted chlorophylls using the example of water plants. J. Exp. Bot..

[70] Vidaković-Cifrek Ž., Tkalec M., Šikić S., Tolić S., Lepeduš H., Pevalek-Kozlina B. (2015). Growth and photosynthetic responses of Lemna minor L. exposed to Cd in combination with Zn or Cu. Arh. Hig. Rada Toksikol..

[71] Petroni O. (1986). Taxonomy of endophytic fungi of aerial plant tissues. Microbiology of Phyllosphere.

[72] Leuchtmann A., Bacon C.W., Schardl C.L., White J.F., Tadych M. (2014). Nomenclatural realignment of *Neotyphodium* species with genus *Epichloë*. Mycologia.

[73] Morgan-Jones G., Gams W. (1982). Notes on hyphomycetes. XLI. An endophyte of *Festuca arundinacea* and the anamorph of *Epichloë typhina*, new taxa in one of two new sections of *Acremonium*. Mycotaxon.

[74] Li C.J., Nan Z.B., Paul V.H., Dapprich P.D., Liu Y. (2004). A new *Neotyphodium* species symbiotic with drunken horse grass (*Achnatherum inebrians*) in China. Mycotaxon.

[75] Moon C.D., Craven K.D., Leuchtmann A., Clement S.L., Schardl C.L. (2004). Prevalence of interspecific hybrids among asexual fungal endophytes of grasses. Mol. Ecol..

[76] Chen L., Li X.Z., Li C.J., Swoboda G.A., Young C.A., Sugawara K., Leuchtmann A., Schardl C.L. (2015). Two distinct Epichloë species symbiotic with *Achnatherum inebrians*, drunken horse grass. Mycologia.

[77] Lembicz M., Górzyńska K., Leuchtmann A. (2010). Choke disease caused by Epichloë bromicola in the grass Agropyron repens in Poland. Plant Dis..

[78] Cagnano G., Roulund N., Jensen C.S., Forte F.P., Asp T., Leuchtmann A. (2019). Large Scale Screening of *Epichloë* Endophytes Infecting Schedonorus pratensis and Other Forage Grasses Reveals a Relation Between Microsatellite-Based Haplotypes and Loline Alkaloid Levels. Front. Plant Sci..

[79] Clay K., Brown V.K. (1997). Infection of *Holcus lanatus* and *H*. *mollis* by *Epichloë* in experimental grasslands. Oikos.

[80] White J.F. (1993). Endophyte-host associations in grasses. XIX. A systematic study of some sympatric species of *Epichloë* in England. Mycologia.

[81] Drake I., White J.F., Belanger F.C. (2018). Identification of the fungal endophyte of *Ammophila breviligulata* (American beachgrass) as *Epichloë amarillans*. Peer J..

[82] Leuchtmann A., Schardl C.L. (1998). Mating compatibility and phylogenetic relationships among two new species of *Epichloë* and other congeneric European species. Mycol. Res..

[83] Charlton N.D., Craven K.D., Afkhami M.E., Hall B.A., Ghimire S.R., Young C.A. (2014). Interspecific hybridization and bioactive alkaloid variation increases diversity in endophytic Epichloë species of *Bromus laevipes*. FEMS Microbiol. Ecol..

[84] McCargo P.D., Iannone L.J., Vignale M.V., Schardl C.L., Rossi M.S. (2014). Species diversity of *Epichloë* symbiotic with two grasses from southern Argentinean Patagonia. Mycologia.

[85] Gentile A., Rossi M.S., Cabral D., Craven K.D., Schardl C.L. (2005). Origin, divergence and phylogeny of *Epichloë* endophytes of native Argentine grasses. Mol. Phylogenet Evol..

[86] Song H., Nan Z., Song Q., Xia C., Li X., Yao X., Xu W., Kuang Y., Tian P., Zhang Q. (2016). Advances in research on Epichloë endophytes in Chinese native grasses. Front. Microbiol..

[87] Ghimire S.R., Rudgers J.A., Charlton N.D., Young C., Craven K.D. (2011). Prevalence of an intraspecific *Neotyphodium* hybrid in natural populations of stout wood reed (*Cinna arundinacea* L.) from eastern North America. Mycologia.

[88] Cabral D., Iannone L.J., Stewart A., Novas M.V. The distribution and incidence of *Neotyphodium* endophytes in native grasses from Argentina and its association with environmental factors. Proceedings of the 6th International Symposium on Fungal Endophytes of Grasses.

[89] Clarke B.B., White J.F., Hurley R.H., Torres M.S., Sun S., Huff D.R. (2006). Endophyte-mediated suppression of dollar spot disease in fine fescues. Plant Dis..

[90] Leuchtmann A., Schardl C.L., Siegel M.R. (1994). Sexual compatibility and taxonomy of a new species of *Epichloë* symbiotic with fine fescue grasses. Mycologia.

[91] Niones J., Takemoto D. (2014). An isolate of *Epichloe festucae*, an endophytic fungus of temperate grasses, has growth inhibitory activity against selected grass pathogens. J. Gen. Plant Pathol..

[92] Craven K.D., Blankenship J.D., Leuchtmann A., Highnight K., Schardl C.L. (2001). Hybrid fungal endophytes symbiotic with the grass *Lolium pratense*. Sydowia.

[93] Gams W., Petrini O.J., Schmidt D. (1990). *Acremonium uncinatum*, a new endophyte in Festuca pratensis. Mycotaxon.

[94] Zhou L., Zhang X., Li C., Christensen M., Zhibiao N. (2015). Antifungal activity and phytochemical investigation of the asexual endophyte of *Epichloe* sp from Festuca sinensis. Science China. Life Sci..

[95] Schardl C.L., Leuchtmann A. (1999). Three new species of *Epichloë* symbiotic with North American grasses. Mycologia.

[96] Leuchtmann A., Oberhofer M. (2013). The *Epichloë* endophytes associated with the woodland grass *Hordelymus europaeus* including four new taxa. Mycologia.

[97] Iannone L.J., Irisarri J.G.N., Mc Cargo P.D., Perez L.I., Gundel P.E. (2015). Occurrence of *Epichloë* fungal endophytes in the sheep-preferred grass *Hordeum comosum* from Patagonia. J. Arid Environ..

[98] Wang X., Qin J., Chen W., Zhou Y., Ren A., Gao Y. (2016). Pathogen resistant advantage of endophyte-infected over endophyte-free *Leymus chinensis* is strengthened by predrought treatment. Eur. J. Plant Pathol..

[99] Moon C.D., Scott B., Schardl C.L., Christensen M.J. (2000). The evolutionary origins of *Epichloë* endophytes from annual ryegrasses. Mycologia.

[100] Latch G.C.M., Christensen M.J., Samuels G.J. (1984). Five endophytes of *Lolium* and *Festuca* in New Zealand. Mycotaxon.

[101] Moon C.D., Miles C.O., Järlfors U., Schardl C.L. (2002). The evolutionary origins of three new Neotyphodium endophyte species from grasses indigenous to the Southern Hemisphere. Mycologia.

[102] Shymanovich T., Charlton N.D., Musso A.M., Scheerer J., Cech N.B., Faeth S.H., Young C.A. (2017). Interspecific and intraspecific hybrid *Epichloë* species symbiotic with the North American native grass *Poa alsodes*. Mycologia.

[103] Tadych M., Ambrose K.V., Bergen M.S., Belanger F.C., White J.F. (2012). Taxonomic placement of *Epichloë poae* sp. nov. and horizontal dissemination to seedlings via conidia. Fungal Divers.

[104] Li W., Ji Y.-L., Yu H.-S., Wang Z.-W. (2006). A new species of *Epichloë* symbiotic with Chinese grasses. Mycologia.

[105] Siegel M.R., Latch G.C.M., Johnson M.C. (1985). *Acremonium* fungal endophytes of Tall Fescue and Perennial ryegrass: Significance and control. Plant Dis..

[106] Semmartin M., Omacini M., Gundel P.E., Hernandez-Agramonte I.M. (2015). Broad-scale variation of fungal-endophyte incidence in temperate grasses. J. Ecol..

[107] Pańka D., Żurek G. (2005). Występowanie grzybów endofitycznych w trawach gazonowych, a ich podatność na stres suszy. Łąkarstwo W Polsce.

[108] Wiewióra B., Prończuk M., Ostrowska A. (2006). Infekcja nasion traw przez endofity w kolejnych latach użytkowania plantacji. Biul IHAR.

[109] Wiewióra B., Prończuk M., Ostrowska A., Żurek G. (2007). Endophyte occurrence in breeding strains of meadow fescue (*Festuca pratensis* Huds.) cv. Pasja. Phytopathol. Pol..

[110] Wiewióra B., Żurek G., Żurek M. (2010). Ocena zasiedlenia przez grzyby endofityczne nasion wybranych mieszanek traw pastewnych dostępnych na rynku krajowym. Biul. IHAR.

[111] Wiewióra B., Żurek G., Pańka D. (2015). Is the vertical transmission of *Neotyphodium lolii* in perennial ryegrass the only possible way to the spread of endophytes?. PLoS ONE.

[112] Malinowski D.P., Belesky D.P. (2000). Adaptations of endophyte-infected cool-season grasses to environmental stresses: Mechanisms of drought and mineral stress tolerance. Crop. Sci..

[113] Żurek M., Ochodzki P., Wiewióra B. (2010). Ocena zawartości ergowaliny w trawach runi wybranych trwałych użytków zielonych na terenie województwa mazowieckiego. Biul. IHAR.

[114] Ravel C., Courty C., Coudret A., Charmet G. (1997). Beneficial effects of *Neotyphodium lolii* on the growth and the water status in perennial ryegrass cultivated under nitrogen deficiency or drought stress. Agronomie.

[115] Arachevaleta M.C., Bacon C.W., Hoveland C.S., Radcliffe D.E. (1989). Effect of the tall fescue endophyte on plant response to environmental stress. Agron. J..

[116] Bacon C.W. (1993). Abiotic stress tolerances (moisture, nutrients) and photosynthesis in endophyte-infected tall fescue. Agric. Ecosyst. Environ..

[117] West C.P., Bacon C.W., White J.F. (1994). Physiology and drought tolerance of endophyte-infected grasses. Biotechnology of Endophytic Fungi of Grasses.

[118] West C.P., Izekor E., Turner K.E., Elmi A.A. (1993). Endophyte effects on growth and persistence of tall fescue along a water–supply gradient. Agron. J..

[119] Elbersen H.W., West C.P. (1996). Growth and water relations of field-grown tall fescue as influenced by drought and endophyte. Gras Forage Sci..

[120] Hill N.S., Pachon J.G., Bacon C.W. (1996). *Acremonium coenophialum* mediated short and long-term drought acclimation in tall fescue. Crop. Sci..

[121] White J.F., Engelke M.C., Morton S.J., Johnson-Cicalese J.M., Ruemmele B.A. (1992). *Acremonium* endophyte effects on tall fescue drought tolerance. Crop. Sci..

[122] Hahn H., McManus M.T., Warnstorff K., Monahan B.J., Young C.A., Davies E., Tapper B.A., Scott B. (2008). *Neotyphodium* fungal endophytes confer physiological protection to perennial ryegrass (*Lolium perenne* L.) subjected to water deficit. Environ. Exp. Bot..

[123] Hunt M.G., Newman J.A. (2005). Reduced herbivore resistance from a novel grass-endophyte association. J. Appl. Ecol..

[124] Gundel P.E., Irisarri J.G.N., Fazio L., Casas C., Pérez L.I. (2016). Inferring field performance from drought experiments can be misleading: The case of symbiosis between grasses and *Epichloë* fungal endophytes. J. Arid. Environ..

[125] Clay K. (1987). Effects of fungal endophytes on the seed and seedling biology of *Lolium perenne* and *Festuca arundinacea*. Oecologia.

[126] Pańka D., Podkówka L., Lamparski R. Preliminary observations on the resistance of meadow fescue (Festuca pratensis Huds.) infected by Neotyphodium uncinatum to diseases and pests and native value. Proceedings of the 5th International Symposium on Neotyphodium/Grass Interactions.

[127] Prończuk M. (2000). Choroby traw–występowanie i szkodliwość w uprawie na nasiona I użytkowaniu trawnikowym. Monogr. i Rozpr. Nauk. IHAR Nr4.

[128] White J.F., Cole G.T. (1985). Endophyte–host associations in forage grasses. III. In vitro inhibition of fungi by *Acremonium coenophialum*. Mycologia.

[129] Siegel M.R., Latch G.C.M. (1991). Expression of antifungal activity in agar culture by isolates of grass endophytes. Mycologia.

[130] Cook R.J., Baker K.F. (1983). The nature and practice of biological control of plant pathogens. Am. Phytopathol. Soc. St. Paul Minn. USA.

[131] Burpee L.L., Bouton J.H. (1993). Effect of eradication of the endophyte *Acremonium coenophialum* on epidemics of Rhizoctonia blight in tall fescue. Plant Dis..

[132] Trevathan L.E. (1996). Performance of endophyte-free and endophyte-infected tall fescue seedlings in soil infected with *Cochliobolus sativus*. Can. J. Plant Pathol..

[133] Blank C.A., Gwinn K.D. (1992). Soilborne seedling diseases of tall fescue: Influence of the endophyte *Acremonium coenophialum*. Phytopathology.

[134] Hume D.E., Quigley P.E., Aldaoud R., Bacon C.W., Hill N.S. (1997). Influence of *Neotyphodium* infection on plant survival of diseased tall fescue and ryegrass. Neotyphodium/Grass Interactions.

[135] Lewis G.C., Bakken A.K., Macduff J.H., Raistrick N. (1996). Effect of infection by the endophytic fungus *Acremonium lolii* on growth and nitrogen uptake by perennial ryegrass (*Lolium perenne*) in flowing solution culture. Ann. Appl. Biol..

[136] Lewis G.C. (2004). Effects of biotic and abiotic stress on the growth of three genotypes of *Lolium perenne* with and without infection by the fungal endophyte *Neotyphodium lolii*. Ann. Appl. Biol..

[137] Hesse U., Schöberlein W., Wittenmayer L., Förster K., Warnstorff K., Diepenbrock W., Merbach W. (2004). Influence of water supply and endophyte infection (*Neotyphodium* spp.) on vegetative and reproductive growth of two *Lolium perenne* L. genotypes. Eur. J. Agron..

[138] Singh L.P., Gill S.S., Tuteja N. (2011). Unraveling the role of fungal symbiosis in plant abiotic stress tolerance. Plant Signal. Behav..

[139] Redman R.R., Sheehan K.B., Stout R.G., Rodriguez R.J., Henson J.M. (2002). Thermotolerance conferred to plant host and fungal endophyte during mutualistic symbiosis. Science.

[140] Rodriguez R.J., Henson J., Van Volkenburgh E., Hoy M., Wright L., Beckwith F., Kim Y.O., Redman R.S. (2008). Stress tolerance in plants via habitat-adapted symbiosis. Int. Soc. Microb. Ecol. J..

[141] Vazquez de Aldana B., Garcia-Criado B., Zabalgogeazcoa I., Garcia-Ciudad A. (1999). Influence of fungal endophyte infection on nutrient element content of tall fescue. J. Plant Nutr..

[142] Monnet F., Vaillant N., Hitmi A., Coudret A., Sallanon H. (2001). Endophytic *Neotyphodium lolii* induced tolerance to Zn stress in *Lolium perenne*. Physiol. Plant..

[143] Monnet F., Vaillant N., Hitmi A., Coudret A., Sallanon H. (2005). Photosynthetic activity of *Lolium perenne* as a function of endophyte status and zinc nutrition. Fuctional. Plant Biol..

[144] Truyens S., Jambon I., Croes S., Janssen J., Weyens N., Mench M., Carleer R., Cuypers A., Vangronsveld J. (2014). The effect of long-term Cd and Ni exposure on seed endophytes of *Agrostis capillaris* and their potential application in phytoremediation of metal-contaminated soils. Inter J. Phytorem..

[145] Żurek G., Wiewióra B., Rybka K., Prokopiuk K. (2021). Different response of perennial ryegrass—Epichloë endophyte symbionts to the elevated concentration of heavy metals in soil.

[146] Hossain Z., Nouri M.Z., Komatsu S. (2012). Plant cell organelle proteomics in response to abiotic stress. J. Proteome. Res..

[147] Ryan R.P., Germaine K., Franks A., Ryan D.J., Dowling D.N. (2008). Bacterial endophytes: Recent developments and applications. FEMS Microbiol. Lett..

[148] Robinson B.H., Lombi E., Zhao F.J., McGrath S.P. (2003). Uptake and distribution of nickel and other metals in the hyperaccumulator Berkheya coddii. New Phytol..

[149] Rosa G., Peralta-Videa J.R., Montes M., Parsons J.G., Cano-Aguilera I., Gardea-Torrresdey J.L. (2004). Cadmium uptake and translocation in tumbleweed (*Salsola kali*), a potential Cd-hyperaccumulator desert plant species. ICP/OES and XAS studies. Chemosphere.

[150] Pulford I.D., Watson C. (2003). Phytoremediation of heavy metal-contaminated land by trees-a review. Environ. Int..

[151] Ma Y., Rajkumar M., Luo Y., Freitas H. (2011). Inoculation of endophytic bacteria on host and non-host plants—effects on plant growth and Ni uptake. J. Hazard. Mater..

[152] Chen L., Luo S., Xiao X., Guo H., Chen J., Wan Y., Li B., Xu T., Xi Q., Rao C. (2010). Application of plant growth-promoting endophytes (PGPE) isolated from *Solanum nigrum* L. for phytoextraction of Cd-polluted soils. Appl. Soil Ecol..

[153] Sheng X., Xia J., Jiang C., He L., Qian M. (2008). Characterization of heavy metal-resistant endophytic bacteria from rape (*Brassica napus*) roots and their potential in promoting the growth and lead accumulation of rape. Environ. Pollut..

[154] Aishwarya S., Venkateswarlu N., Chandra Mouli K., Vijaya T. (2014). Role of endophytic fungi in the restoration of heavy metal contaminated soils. Indo Am. J. Pharm. Res..

[155] Zhang Y., He L., Chen Z., Zhang W., Wang Q., Qian M., Sheng X. (2011). Characterization of lead-resistant and ACC deaminase-producing endophytic bacteria and their potential in promoting lead accumulation of rape. J. Hazard. Mater..

[156] Weyens N., Truyens S., Dupae J., Newman L., Taghavi S., Lelie D., Carleer R., Vangronsveld J. (2010). Potential of the TCE degrading endophyte *Pseudomonas putida* W619-TCE to improve plant growth and reduce TCE phytotoxicity and evapotranspiration in poplar cuttings. Environ. Pollut..

[157] Germaine K.J., Keogh E., Ryan D., Dowling D. (2009). Bacterial endophyte-mediated naphthalene phytoprotection and phytoremediation. FEMS Microbiol. Lett..

[158] Sheng X., Chen X., He L. (2008). Characteristics of an endophytic pyrene-degrading bacterium of Enterobacter sp. from *Allium macrostemon* Bunge. Int. Biodeterior Biodegrad..

[159] Shin M., Shim J., You Y., Myung H., Bang K., Cho M., Kamala-Kannan S., Oh B. (2011). Characterization of lead resistant endophytic *Bacillus* sp. MN3-4 and its potential for promoting lead accumulation in metal hyperaccumulator *Alnus firma*. J. Hazard. Mater..

[160] Hamayun M., Sumera A.K., Iqbal I., Ahmad B., Lee I. (2010). Isolation of a gibberellin producing fungus (*Penicillium* sp. MH7) and growth promotion of crown daisy (*Chrysanthemum coronarium*). J. Microbiol. Biotechnol..

[161] Saravanan V.S., Madhaiyan M., Thangaraju M. (2007). Solubilization of zinc compounds by the diazotrophic, plant growth promoting bacterium *Gluconacetobacter diazotrophicus*. Chemosphere.

[162] Yousaf S., Andria V., Reichenauer T.G., Smalla K., Sessitsch A. (2010). Phylogenetic and functional diversity of alkane degrading bacteria associate with Italian ryegrass (*Lolium multiflorum*) and Birdsfoot trefoil (*Lotus corniculatus*) in a petroleum oil-contaminated environment. J. Hazard. Mater..

[163] Barac T., Taghavi S., Borremans B., Provoost A., Oeyen L., Colpaert J.V., Vangronsveld J., van der Lelie D. (2004). Engineered endophytic bacteria improve phytoremediation of water-soluble, volatile, organic pollutants. Nat. Biotechnol..

[164] Gunjal A.B., Waghmode M.S., Patil N.N., Kapadnis B.P. (2018). Endophytes and their role in phytoremediation and biotransformation process. Chapter 15 in Microbial Biotechnology in Environmental Monitoring and Cleanup, Advances in Environmental Engineering and Green Technologies.

[165] Mannisto M.K., Tiirola M.A., Puhakka J.A. (2001). Degradation of 2,3,4,6-tetrachlorophenol at low temperature and low dioxygen concentrations by phylogenetically different groundwater and bioreactor bacteria. Biodegradation.

[166] Van A., Yoon J.M., Schnoor J.L. (2004). Biodegradation of Nitro-Substituted explosives 2,4,6-Trinitrotoluene, Hexahydro-1,3,5-Triazine and Octahydro-1,3,5,7-Tetranitro-1,3,5-Tetrazocine by a phytosimbotic Methylobacterium spp. associated with poplar tissues (Populus deltoids x nigra DN34). Appl. Environ. Microbiol..

[167] Soleimani M., Hajabbasi M.A., Afyuni M., Mirlohi A., Borggaard O.K., Holm P. (2010). Effect of endophytic fungi on cadmium tolerance and bioaccumulation by *Festuca arundinacea* and *Festuca pratensis*. Int. J. Phytorem.

[168] Li H.Y., Li D.W., He C.M., Zhou Z.P., Mei T., Xu H.M. (2012). Diversity and heavy metal tolerance of endophytic fungi from six dominant plant species in a Pb-Zn mine wasteland in China. Fungal Ecol..

[169] Li X., Li W., Chu L., White J.F., Xiong Z., Li H. (2016). Diversity and heavy metal tolerance of endophytic fungi from *Dysphania ambrosioides,* a hyperaccumulator from Pb-Zn contaminated soils. J. Plant Interact..

[170] Zhang X., Fan X., Li C., Nan Z. (2010). Effects of cadmium stress on seed germination, seedling growth and antioxidative enzymes in *Achnatherum inebrians* plants infected with a *Neotyphodium* endophyte. Plant Growth Regul..

[171] Zhang X., Li C., Nan Z. (2010). Effects of cadmium stress on growth and anti-oxidative systems in *Achnatherum inebrians* symbiotic with a *Neothypodium gansuensee*. J. Hazard. Mater..

[172] Zhang X., Li C., Nan Z. (2012). Effects of cadmium stress on seed germination and seedling growth of *Elymus dahuricus* infected with the *Neotyphodium* endophyte. Sci. China Life Sci..

[173] Soleimani M., Afyuni M., Hajabbasi M.A., Farshid Nourbakhsh F., Sabzalian M.R., Christensen J.H. (2010). Phytoremediation of an aged petroleum contaminated soil using endophyte infected and non-infected grasses. Chemosphere.

[174] Zamani N., Sabzalian M.R., Khoshgoftarmanesh A., Afyuni M. (2015). *Neotyphodium* endophyte changes phytoextraction of Zinc in *Festuca arundinacea* and *Lolium perenne*. Int. J. Phytorem.

[175] Malinowski D.P., Belesky D.P. (1999). Tall fescue aluminum tolerance is affected by *Neotyphodium coenophialum* endophyte. J. Plant Nutr..

[176] Zaurov D.E., Bonos S., Murphy J.A., Richardson M., Belanger F.C. (2001). Endophyte infection can contribute to aluminium tolerance in fine fescues. Crop. Sci..

[177] Benavides M.P., Gallego S.M., Tomaro M.L. (2005). Cadmium toxicity in plants. Braz. J. Plant Physiol..

[178] Zhang F.Q., Zhang H.X., Wang G.P., Xu L.L., Shen Z.G. (2009). Cadmium induced accumulation of hydrogen peroxide in the leaf apoplast of *Phaseolus aureus* and *Vicia sativa* and the roles of different antioxidant enzymes. J. Hazard. Mater..

[179] Ren A., Gao Y., Zhang L., Xie F. (2006). Effect of cadmium on growth parameters of endophyte-infected and endophyte-free ryegrass. J. Plant Soil Sci..

[180] Dennis S.B., Allen V.G., Saker K.E., Fontenot J.P., Ayad J.Y.M., Brown C.P. (1998). Influence *of Neotyphodium coenophialum* on copper concentration in tall fescue. J. Anim. Sci..

[181] Malinowski D.P., Zuo H., Belesky D.P., Alloush G.A. (2004). Evidence for copper binding by extracellular root exudates of tall fescue but not perennial ryegrass infected with *Neotyphodium* spp. Endophytes. Plant Soil.

[182] Mirzahosseini Z., Shabani L., Sabzalian M.R., Sharifi T.M. (2014). Effect of *Neotyphodium* endophyte symbiosis on growth, nickel uptake and photosynthetic pigments in two genotypes of tall fescue. J. Plant Process Funct..

[183] Latch C.G.M. (1993). Physiological interactions of endophytic fungus and their host. Biotic stress tolerance imparted to grasses by endophytes. Agric. Ecosyst Environ..

[184] Ren A., Li C., Gao Y. (2011). Endophytic fungus improves growth and metal uptake *of Lolium arundinaceum* Darbyshire ex. Schreb. Inter J. Phytorem.

[185] Kuldau G., Bacon C.W. (2008). *Clavicipitaceous* endophytes: Their ability to enhance resistance of grasses to multiple stresses. Biol. Control.

[186] Khan Z., Doty S.L. (2011). Endophyte-assisted phytoremediation. Curr. Top Plant Biol..

[187] Harmens H., Gusmao N.G.C.P.B., Den Hartog P.R., Verkleij J.A.C., Ernst W.H.O. (1993). Uptake and transport of Zn in Zn-sensitive and Zn-tolerant Silene _ulgaris. J. Plant Physiol..

[188] Longnecker N.E., Robson A.D., Robson A.D. (1993). Distribution and transport of Zn in plants. Zn in Soils and Plants.

[189] Pitzschke A. (2016). Developmental peculiarities and seed-borne endophytes in quinoa: Omnipresent robus bacilli contribute to plant fitness. Front. Microbiol..

